# Initiatives to address leprosy as a human rights issue through the mandate of UN Special Rapporteur: Achievements and challenges

**DOI:** 10.1371/journal.pntd.0010201

**Published:** 2022-03-17

**Authors:** Takahiro Nanri

**Affiliations:** Sasakawa Health Foundation, Tokyo, Japan; Shandong Provincial Institute of Dermatology and Venereology, CHINA

## Abstract

Leprosy, or Hansen’s disease, is one of the oldest infectious diseases in the world. It has long been associated with stigma and discrimination, but only in recent years has this aspect been formally recognized by the international community as a human rights issue. The UN Human Rights Council first adopted a resolution on leprosy in 2008, and this was later followed by a UN General Assembly resolution in 2010. Nonbinding principle and guidelines on elimination of discrimination against persons affected by leprosy and their family members accompanied the 2010 resolution, but these have yet to be fully implemented. In 2017, the Human Rights Council appointed a Special Rapporteur on leprosy to investigate the extent to which the principles and guidelines have been implemented, and her term was extended for a further 3 years in 2020. Considering the proper implementation of the principles and guidelines to be key to eliminating the discrimination that persons affected by leprosy and their families face in various parts of the world, this paper looks at the contribution the Special Rapporteur can make. Based on an assessment of her activities to date, it concludes that the Special Rapporteur has actively worked to build networks with persons affected by leprosy and related organizations and gain their trust, but has faced challenges in organizing official country visits. It goes on to analyze what sort of legacy the Special Rapporteur should aim to leave behind after completing her second term and how she can go about doing so in the time remaining. To this end, it makes 5 suggestions: (1) gather information systematically on the actual situation of discrimination; (2) compile a collection of success stories; (3) ensure that there is consistency between legally binding international covenants and treaties and the principles and guidelines; (4) present proposals for concrete actions that can be taken after the Special Rapporteur’s second term ends; and (5) initiate a feasibility study on creating an “index” and “indicators” to measure the current status of stigma and discrimination and the extent to which the principles and guidelines have been implemented.

## Introduction

Leprosy, also known as Hansen’s disease, is an infectious disease caused by the bacillus *Mycobacterium leprae*. Once incurable, leprosy is now treatable with multidrug therapy (MDT). But if treatment is delayed, the disease, which mainly affects the skin and peripheral nerves, can result in lifelong disability. Despite medical advances, outdated notions of the disease persist, meaning that persons who have been cured and even their family members face severe discrimination. For example, the World Health Organization (WHO) states that one or more instances of discrimination were reported in 51 countries in 2020 [1]. In addition, according to data compiled by the International Federation of Anti-Leprosy Associations (ILEP), there are 139 discriminatory laws associated with leprosy at least in 24 countries [2].

Why does such discrimination still exist? Without cure for much of its long history, leprosy’s progressively disfiguring effects fears that it was highly contagious or even that it was divine punishment shaped perceptions of the disease. Second, after Norwegian doctor Gerhard Armauer Hansen’s discovery of *M*. *leprae* revealed leprosy to be an infectious disease caused by a bacillus, the introduction of compulsory segregation in various parts of the world reinforced the idea that already existed that people with leprosy were to be excluded from society, an image that has lingered in people’s minds.

Therefore, a “social” as well as a “medical” approach is essential in dealing with leprosy. Based on this, WHO has recently issued its “Global Leprosy Strategy 2021–2030” [3], which sets “Combat stigma and ensure human rights” as one of the 4 pillars. This means that all those who engage in leprosy services at any stages, be they doctors or health workers, are required to pay attention not only to the care of disease and disabilities but also to the problems of stigma and discrimination that persons affected by leprosy encounter.

Only recently leprosy has been formally recognized by the international community as a human rights issue. The United Nations Human Rights Council (UNHRC) first adopted a resolution on “Elimination of discrimination against persons affected by leprosy and their family members” in 2008. In 2010, the United Nations General Assembly unanimously approved its own resolution, which noted with appreciation principles and guidelines for the elimination of discrimination against persons affected by leprosy and their family members that provide governments with a roadmap on how to address the issue. More recently, the UNHRC appointed a United Nations Special Rapporteur on the elimination of discrimination against persons affected by leprosy and their family members in September 2017 for a 3-year term, and, in July 2020, her term was extended by 3 years.

Shigeki Sakamoto, who drafted the principles and guidelines as a member of the Advisory Committee of the UNHRC, described the document’s significance as follows: “First, international standards of human rights have been established for persons affected by leprosy. Second, it clarifies that persons affected by leprosy are entitled to human rights under international law [4].” However, since governments are not forced to comply with them, the issue is how the principles and guidelines will actually be implemented and how they will bring an end to leprosy-related discrimination.

### The UN resolution and the principles and guidelines

To date, the UNHRC has adopted resolutions on the elimination of discrimination against persons affected by leprosy and their family members on 6 occasions (2008, 2009, 2010, 2015, 2017, and 2020) as shown in Table 1 below. The Nippon Foundation, a Japanese nonprofit organization, played a central role in the process under its chairman Yohei Sasakawa, collaborating closely with the Japanese government, organizations of persons affected by leprosy, researchers, and others to help lay the groundwork for these resolutions to be adopted.

**Table 1:** Overview of HRC Resolutions.

Resolution 8/13 [5]	Resolution 12/7 [6]	Resolution 15/10 [7]	Resolution 29/5 [8]	Resolution 35/9 [9]	Resolution 44/6 [10]
8th session of HRC (June 2–18, 2008)	12th session of HRC (September 14–October 2, 2009	15th session of HRC (September 13–October 1, 2010)	29th session of HRC (June 15–July 3, 2015)	35th session of HRC (June 6–23, 2017)	44th session of HRC (June 30–July 17, 2020)
States that leprosy should be recognized as a human rights issue and requests the HRC Advisory Committee to formulate a draft set of P&G for the elimination of leprosy-related discrimination and submit it by September 2009.	Requests that the OHCHR collects the views of governments, UN agencies, NGOs, and representatives of persons affected by leprosy on P&G and submits a final draft reflecting these views to the 15th session of the HRC.	Approves the final draft of P&G and invites the UN General Assembly to consider the issue of leprosy-related discrimination.	Mandates the Advisory Committee to review the implementation status of P&G and submit a report containing suggestions for their wider dissemination and more effective implementation to the 35th session of the HRC.	Appoints a Special Rapporteur on elimination of discrimination against persons affected by leprosy and their family members for a 3-year term.	Extends the term of the Special Rapporteur for 3 years.

(Source: Prepared by the author based on the past resolutions).

HRC, Human Rights Council; NGO, nongovernmental organization; OHCHR, Office of the High Commissioner for Human Rights; P&G, principles and guidelines.

The principles and guidelines [11] that accompanied the Resolution in 2010 are key to eliminating the stigma and discrimination related to leprosy. First, as principles, persons affected by leprosy and their family members should be treated as people with dignity and are entitled, on an equal basis with others, to all the human rights and fundamental freedoms proclaimed in the Universal Declaration of Human Rights, as well as in other relevant international human rights instruments. Further, they should not be discriminated against on the grounds of having or having had leprosy, and have the same rights as everyone else with respect to the following: (1) marriage, family, and parenthood; (2) full citizenship and obtaining identity documents; (3) the right to work; (4) education and participation in training programs; (5) the full realization of their dignity and self-worth; and (6) the right to be involved in decision-making processes regarding policies and programs that directly concern their lives. As for the guidelines, governments are encouraged to address 14 areas, which are summarized in Table 2.

**Table 2:** Gist of the principles and guidelines.

	Principles
	Persons affected by leprosy and their family members should be treated as people with dignity and are entitled to all human rights and fundamental freedoms.
	**Guidelines**
1	States should promote, protect, and ensure the full realization of all human rights and fundamental freedoms for all persons affected by leprosy and their family members without discrimination on the grounds of leprosy.
2	States should recognize that all persons are equal before and under the law.
3	States should pay special attention to the promotion and protection of the rights of women, children, and members of other vulnerable groups who have or have had leprosy.
4	States should, where possible, support the reunification of families separated as a result of past policies and practices relating to leprosy.
5	States should promote the enjoyment of the same rights for persons affected by leprosy and their family members as for everyone else, allowing their full inclusion and participation in the community.
6	States should secure the rights of participation in political life.
7	States should encourage and support opportunities for vocational training and employment.
8	States should promote equal access to education.
9	States should remove discriminatory language from governmental publications.
10	State should promote equal access to public places, public transport, cultural and recreational facilities, and places of worship.
11	States should provide persons affected by leprosy with free or affordable healthcare of a standard on a par with that provided persons with other diseases.
12	States should recognize the rights of persons affected by leprosy to an adequate standard of living, and should take appropriate steps to safeguard and promote that right.
13	States should raise awareness and promote respect for human rights and dignity.
14	States should create a committee of stakeholders to develop, implement, and follow up on activities relating to human rights.

(Source: Prepared by the author based on the Principles and Guidelines [11]).

### Activities of the UN Special Rapporteur on leprosy

The Special Rapporteur has submitted annual thematic reports to the UNHRC since 2018. In her first report [12], she noted that discrimination related to leprosy still exists at the macro level of national policy and laws, at the micro level of community and family, and in between within institutions and organizations (relating to health, education, employment, etc.). The report stressed that, in order to eliminate such structural discrimination, it is important to involve those affected by the disease.

In her 2019 report focusing on “wrongful stereotyping and structural violence against women and children affected by leprosy [13],” she at first raises the issue of the lack of reliable data on how stereotypes and practices impact the lives of persons affected by leprosy. Then she emphasizes how persons affected have historically been “dehumanized.”

The theme of the 2020 report is a “Policy framework for rights-based action plans [14],” and the report lists a number of initiatives as a final recommendation, including the following: (1) respect the rights of persons affected; (2) ensure the participation of persons affected and create a system of governance to guarantee it; (3) collect data on the actual situation of discrimination; (4) construct monitoring systems for human rights violations; (5) allocate an appropriate budget at the national and state level; (6) promote international cooperation; and (7) pursue initiatives linked to the sustainable development goals.

In her latest thematic report submitted to the UNHRC in 2021, the Special Rapporteur focuses on the disproportionate impact of COVID-19 on persons affected by leprosy and their family members [15]. According to the report, persons affected by leprosy, who have already been excluded from society, are severely impacted by COVID-19 and face various problems, including unemployment, loss of livelihoods, and difficulty in accessing existing safety nets. Taking into account the fact that persons affected are among society’s most vulnerable members, she recommends that governments should immediately take both short- and medium-term measures to respond to the situation.

### Achievements and challenges

One of the Special Rapporteur’s achievements to date is the way she has built links with persons affected by leprosy and related organizations around the world and gained their trust. Furthermore, the presence of the Special Rapporteur is encouraging networking among persons affected by leprosy to progress and is also contributing to their empowerment.

On the other hand, there have also been challenges. During her first term, the Special Rapporteur was scheduled to make 6 official country visits in 3 years, but only realized visits to 2 target countries, Brazil and Japan, which remains the case to date. One can speculate as to the reasons: (1) many governmental officials do not see leprosy as a human rights issue; (2) the Office of the High Commissioner for Human Rights has not prioritized leprosy compared to other human rights issues, and its support for the Special Rapporteur has been insufficient; (3) the global leprosy community is relatively small and not influential enough to exert pressure on target countries to accept visits; (4) initial approaches to target countries got off to a slow start because this was a newly established mandate; and (5) the coronavirus pandemic that took hold in the latter stages of the Special Rapporteur’s term made country visits impossible.

### What role is expected of the Special Rapporteur?

Now into her second term, the question is what the Special Rapporteur can do in the time remaining through 2023, and what legacy she can leave behind. Ideally, she will be able to establish a pathway to encourage countries to properly implement the principles and guidelines.

This paper makes 5 suggestions:

1. Gather information on the actual situation of discrimination. Work with the various different stakeholders involved with leprosy to summarize in a systematic way the circumstances under which persons affected by leprosy are currently discriminated against and the nature of that discrimination. As information currently exists only in fragmentary pieces, in order to resolve the issue of discrimination, it is necessary to clearly identify the kinds of discrimination that actually exist as well as the real socioeconomic circumstances of the persons affected.
2. Compile a collection of success stories for dealing with stigma and discrimination and work with researchers in each region to document examples that incorporate detailed information and expert analysis, in close cooperation with communities of persons affected by leprosy. These case studies should prove of value to governments in drawing up concrete measures, such as promoting participation of persons affected in decision-making processes that affect them, abolishing discriminatory laws, and developing a pension system for persons affected.
3. Prepare a paper in collaboration with international legal scholars showing that the principles and guidelines are consistent with legally binding international laws and treaties. Should such a document be published in the name of the Special Rapporteur, it will carry a weight of authority that governments will not be able to ignore.
4. Present proposals for concrete actions to be taken after the Special Rapporteur’s second term ends. For example, the UNHRC is currently considering consolidating the work of Special Rapporteurs, so there are options such as encouraging other Special Rapporteurs, such as the Special Rapporteur on the right to health, to give due consideration to leprosy.
5. Initiate a feasibility study on the possibility of creating an “index” and “indicators” to measure the current status of stigma and discrimination and the extent to which the principles and guidelines have been implemented. The index would be used to measure the situation from the perspective of persons affected by leprosy and their families, and show a way forward. For example, developing a tool for leprosy equivalent to “The People Living with HIV (PLHIV) Stigma Index [16]” would help grasp the actual situation of discrimination. Indicators would aim at measuring what steps countries have taken against leprosy in, for example, the 14 areas listed above in the principles and guidelines. Such an index and indicators could be used as resources for the formulation of policies and programs to eliminate stigma and discrimination associated with leprosy in member States. The Special Rapporteur can at least start initiating the process during her term, given the time it would take to carry this out.

Of course, the Special Rapporteur cannot implement all the above suggestions alone, so it is essential to establish a more strategic and specific mechanism of cooperation among key stakeholders for supporting her initiatives. Support from the global leprosy community could even include convincing States implement recommendations made by the Special Rapporteur in her latest report to respond to COVID-19, given that their reactions are apathetic. Furthermore, support for her should extend beyond the leprosy community and come from the neglected tropical disease (NTD) community as a whole. Given that the problems persons affected by leprosy face have much in common with problems that persons affected by other NTDs face, transferring experiences and lessons learnt from leprosy can be applicable to eliminate stigma and discrimination associated with other NTDs.

Finally, it is true that we still have a long journey to eliminate stigma and discrimination against persons affected by leprosy and their family members. In the process, the principles and guidelines should be considered as an important tool to achieve that goal. The role of the Special Rapporteur in seeking to have States properly implement them, and in continuously working on mainstreaming leprosy as a human rights issue, is crucial.

## References

[1] World Health Organization. Global Leprosy Update, 2020. Wkly Epidemiol Rec. 2020;95:417–44-0 [cited 2021 Oct 20]. Available from: https://www.who.int/publications/i/item/who-wer9636-421-444

[2] International Federation of Anti-Leprosy Association (ILEP). Updated list of discriminatory laws [cited 2022 Feb 3]. Available from https://ilepfederation.org/updated-list-of-discriminatory-laws/.

[3] World Health Organization. Towards Zero Leprosy. Global Leprosy(Hansen’s disease) Strategy 2021–2030. 2021 [cited 2021 Oct 20]. Available from: https://www.who.int/publications/i/item/9789290228509.

[4] Sakamoto S. Principles and guidelines for the elimination of stigma and discrimination against persons affected by leprosy and their family members. Kansai University Review of Law and Politics. 2011;61(3):754–805. Faculty of Law, Kansai University. Japanese.

[5] United Nations Human Rights Council. Resolution 8/13. Elimination of discrimination against persons affected by leprosy and their family members, A/HRC/RES/8/13. 2008 June 18 [cited 2022 Feb 1]. Available from: https://ap.ohchr.org/Documents/E/HRC/resolutions/A_HRC_RES_8_13.pdf.

[6] United Nations General Assembly. Promotion and protection of all human rights, civil, political, economic, social and cultural rights, including the right of development. Resolution adopted by Human Rights Council. 12/7. Elimination of discrimination against persons affected by leprosy and their family members, A/HRC/RES/12/7. 2009 October 12 [cited 2022 Feb 1]. Available from: https://documents-dds-ny.un.org/doc/RESOLUTION/GEN/G09/165/64/PDF/G0916564.pdf?OpenElement.

[7] United Nations General Assembly. Resolution adopted by Human Rights Council. 15/10. Elimination of discrimination against persons affected by leprosy and their family members, A/HRC/RES/15/10. 2010 October 6 [cited 2022 Feb 1]. Available from: https://documents-dds-ny.un.org/doc/UNDOC/GEN/G10/166/40/PDF/G1016640.pdf?OpenElement.

[8] United Nations General Assembly. Resolution adopted by Human Rights Council on 2 July 2015. 29/5. Elimination of discrimination against persons affected by leprosy and their family members, A/HRC/RES/29/5. 2015 July 21 [cited 2022 Feb 1]. Available from: https://documents-dds-ny.un.org/doc/UNDOC/GEN/G15/161/61/PDF/G1516161.pdf?OpenElement.

[9] United Nations General Assembly. Resolution adopted by Human Rights Council on 22 June 2017. 35/9. Elimination of discrimination against persons affected by leprosy and their family members, A/HRC/RES/35/9. 2017 July 12 [cited 2022 Feb 1]. Available from: https://documents-dds-ny.un.org/doc/UNDOC/GEN/G17/185/03/PDF/G1718503.pdf?OpenElement.

[10] United Nations General Assembly. Resolution adopted by Human Rights Council on 16 July 2020. Elimination of discrimination against persons affected by leprosy and their family members, A/HRC/RES/44/6. 2020 July 10 [cited 2022 Feb 1]. Available from: https://undocs.org/en/A/HRC/RES/44/6.

[11] United Nations General Assembly. Draft set of principles and guidelines for the Elimination of discrimination against persons affected by leprosy and their family members, A/HRC/15/30. 2010 August 12 [cited 2021 Oct 20]. Available from: https://www.mofa.go.jp/files/000078250.pdf.

[12] United Nations General Assembly. Report of the Special Rapporteur on the elimination of discrimination against persons affected by leprosy and their family members, A/HRC/38/42. 2018 May 25 [cited 2021 Oct 20]. Available from: https://documents-dds-ny.un.org/doc/UNDOC/GEN/G18/139/68/PDF/G1813968.pdf?OpenElement.

[13] United Nations General Assembly. Stigmatization as dehumanization: wrongful stereotyping and structural violence against women and children affected by leprosy-Report of the Special Rapporteur on the elimination of discrimination against persons affected by leprosy and their family members, A/HRC/41/47. 2019 April 16 [cited 2021 Oct 20]. Available from: https://documents-dds-ny.un.org/doc/UNDOC/GEN/G19/108/91/PDF/G1910891.pdf?OpenElement.

[14] United Nations General Assembly. Policy framework for rights-based action plans-Report of the Special Rapporteur on the elimination of discrimination against persons affected by leprosy and their family members, A/HRC/44/46. 2020 April 27 [cited 2021 Oct 20]. Available from: https://undocs.org/en/A/HRC/44/46

[15] United Nations General Assembly. Disproportionate impact of the coronavirus disease (COVID-19) pandemic on persons affected by leprosy and their family members: root causes, consequences and the way to recovery–Report of the Special Rapporteur on the elimination of discrimination against persons affected by leprosy and their family members, A/HRC/47/29. 2020 April 8 [cited 2021 Oct 20]. Available from: https://undocs.org/A/HRC/47/29.

[16] Friedland B, Gottert A, Hows J, Baral S, Sprague L, Nyblade L, et al. The People Living with HIV Stigma Index 2.0: generating critical evidence for change worldwide. Wolters Kluwer Health, Inc. 2020 [cited 2021 Oct 20]. Available from: https://pubmed.ncbi.nlm.nih.gov/32881790/. 10.1097/QAD.0000000000002602 PMC11758485 32881790

